# Chiropractic in Global Health and wellbeing: a white paper describing the public health agenda of the World Federation of Chiropractic

**DOI:** 10.1186/s12998-018-0194-y

**Published:** 2018-07-17

**Authors:** Michele Maiers, Mustafa Agaoglu, Richard Brown, Christopher Cassirer, Kendrah DaSilva, Reidar P. Lystad, Sarkaw Mohammad, Jessica J. Wong

**Affiliations:** 1 Northwestern Health Sciences University, 2501 W 84th St, Bloomington, MN 55431 USA; 2 Bahçeşehir University Health Sciences School of Chiropractic, No:10, Gayrettepe, 34353 Beşiktaş, Istanbul, Turkey; 3 World Federation of Chiropractic, 160 Eglinton Avenue East Suite 601, Toronto, ON M4P 3B5 Canada; 4 Northwestern Health Sciences University, 2501 W 84th St, Bloomington, MN 55431 USA; 5 Chiropractic Association of South Africa, Crossway Office Park, 240 Lenchen Ave, Centurion, Pretoria, 0157 South Africa; 6 Australian Institute of Health Innovation, Macquarie University, Sydney, Australia; 7 Hillcrest Spinal Centre, 174 Cambridge Road Hillcrest Hamilton, Hamilton, 3216 New Zealand; 8 Canadian Memorial Chiropractic College, 6100 Leslie Street, Toronto, ON M2H 3J1 Canada

**Keywords:** Chiropractic, Public health, Ageing, Opioid, women’s health, children’s health

## Abstract

The World Federation of Chiropractic supports the involvement of chiropractors in public health initiatives, particularly as it relates to musculoskeletal health. Three topics within public health have been identified that call for a renewed professional focus. These include healthy ageing; opioid misuse; and women’s, children’s, and adolescents’ health. The World Federation of Chiropractic aims to enable chiropractors to proactively participate in health promotion and prevention activities in these areas, through information dissemination and coordinated partnerships. Importantly, this work will align the chiropractic profession with the priorities of the World Health Organization. Successful engagement will support the role of chiropractors as valued partners within the broader healthcare system and contribute to the health and wellbeing of the communities they serve.

## Background

Public Health is defined as the art and science of preventing disease, prolonging life, and promoting health through organized efforts of society [1]. Public health initiatives involve comprehensive approaches to promote the well-being of a community across multiple sectors, including healthcare professions, funders, governments, non-governmental organizations, and other stakeholders. In an era where both medical costs and years lived with chronic disease are increasing, [2] calls have been made for closer collaboration between public health officials and healthcare providers [3]. The potential contribution of many providers, including chiropractors and other healthcare professionals, is often overlooked. This results in an underutilized workforce for health promotion, chronic disease prevention, primary care and implementation of public health initiatives. To help address this gap, it is important for the chiropractic profession to identify priority areas of focus, and clearly articulate plans for engagement in public health. Coordinating this on an international scale allows an opportunity for effective use of resources and consistent application of the profession’s expertise, particularly in musculoskeletal health.

The World Federation of Chiropractic (WFC) is a global non-profit organization representing national chiropractic associations of chiropractors in over 90 countries. The organization serves as a global voice for the chiropractic profession, promoting high standards of conduct, research, education, and practice. The WFC is the only chiropractic organization with non-governmental status with the World Health Organization (WHO) and is committed to supporting public health initiatives and advancing spinal health and well-being through these activities. The WFC has supported several WHO initiatives over the past two decades, including its tobacco-free initiative and the Global Strategy on Diet, Physical Activity and Health. Since 2012, The WFC also organizes and promotes World Spine Day, raising awareness about spinal disorders as a part of the Bone and Joint Decade’s Action Week.

The WFC’s Public Health Committee has committed to an expanded agenda that focuses on three new priority areas of public health: healthy ageing; opioid overuse and misuse; and women’s, children’s, and adolescents’ health. These were chosen for their alignment with WHO priorities, and the chiropractic profession’s ability to uniquely contribute to each through the lens of musculoskeletal health. The goal is to enhance the ability for chiropractors to actively engage in health promotion activities in alignment with WHO priority areas and pursue collaborative work to increase global attention on these important public health issues. As a first step, the WFC will focus on providing key strategies that chiropractors in primary care settings can focus on bridging their work in primary care and population health. The WFC has developed position statements and proposed public health strategies for each priority area, as described below.

## Priority area: Healthy ageing

### Context

The world’s population is ageing rapidly [4]. The number of people aged 60 years and over is expected to double during the first half of the twenty-first century [5]. This demographic shift is not transient, but enduring [4]. Resulting social transformations will be significant, with profound implications for nearly all sectors of society [4, 5]. There is a clear need for a comprehensive public health approach to population ageing – an approach that responds to the capacities and aspirations of older adults and the changing contexts in which they function [6].

Since older adults experience a range of health and functional states, patient-centered healthcare must be tailored to meet the unique needs of this special population. Healthcare providers should accommodate the evolving expectations of older people, who are increasingly looking for innovative alternatives to traditional retirement and healthcare to meet their individual needs. This includes, but is not necessarily limited to, prevention, early detection, and control of chronic conditions; reversing or slowing declines in functional capacity; managing advanced chronic conditions; and end-of-life care. The WFC recognizes opportunities to contribute to global health by prioritizing care that focuses on health promotion of the ageing population.

### Healthy ageing and chiropractic practice

Chronic non-communicable diseases account for most of the burden of disease among older adults [7]. Therefore, primary and secondary prevention is a vital component of geriatric healthcare. Musculoskeletal conditions are a leading contributor to non-communicable burden of disease, predominantly low-back pain and osteoarthritis [7]. Physical activity is key in the prevention, treatment, and management of most chronic conditions affecting older adults, including musculoskeletal complaints [8–10]. Chiropractors should consider prescribing exercise, with or without manual therapy, for spine care in older adults. Such approaches are supported by an evidence-based framework, which includes clinical practice guidelines [11–13].

Fall-related injury is major public health concern that is associated with very high morbidity and mortality in older adults [14]. There is a vast array of risk factors for falls in older adults, including physical frailty, muscle weakness, unsteady gait and balance, and impaired cognition [15]. To reduce the risk of falls, older adults should be prescribed exercise that maintains and improves strength, balance and mobility [16–19]. Exercise recommendations should be in accordance with current evidence-based physical activity guidelines for older adults, [20] and with dose, intensity, and frequency tailored to each individual patient.

Older adults are likely to experience multiple chronic conditions at the same time, [5, 21] and may be concurrently receiving care from multiple healthcare providers. Integration and collaboration is paramount for healthcare providers who deliver services to older adults. Clear communication and collaboration between chiropractors and other health care provers as members of an older adult’s healthcare team is recommended to improve health outcomes and care experiences.

Access to chiropractic care may present a barrier for older adults, especially in low- and middle-income countries. Although good quality data is scarce, people aged 65 years and over are arguably underserviced by the chiropractic profession, [22] a phenomenon likely to vary considerably by geographic location and socioeconomic status. Potential barriers to access include cost of services, restricted transportation, lack of awareness of services offered, failure to seek services due to lack of perceived benefit, and inadequate skills and knowledge among providers to treat this patient population [5]. The WFC encourages practitioners to increasingly deliver services where older adults live (e.g. retirement communities, residential aged care facilities, and domiciliary assisted living residences), improve communication with patients who have sensory impairments (e.g. hearing, vision), enhance ease of access and mobility within chiropractic clinics, and work with other healthcare providers and community services to help reduce the known barriers for older adults receiving care.

The WFC believes the chiropractic profession can play a role in a comprehensive public health approach to population ageing. Engagement in healthy ageing initiatives, utilization of evidence-informed resources and continued professional development will improve and broaden chiropractors’ knowledge, expertise, and competence in providing care for older adults.

## Priority area: Opioid misuse

### Context

Opioid misuse is a complex public health crisis, and has devastating consequences for individuals, families, and communities. In 2015, approximately 29.5 million people worldwide suffered from problematic drug use and related disorders, including dependence [23]. Of these, it is estimated that 13.5 million people used and abused opioids [24]. Opioids account for 70% of the negative health impact associated with drug use disorders globally, and are considered the most harmful drug type [25]. Approximately 69,000 people worldwide die from opioid overdose each year, with a large toll of overdose deaths in the United States and Canada [26]. In addition to overdose-related deaths, high dose opioid therapy is associated with considerable morbidity, including addiction, drug toxicity, falls, fractures, and traffic injuries [27–31].

The escalation of opioid overdoses and related deaths coincides with the increased use of opioids for the management of chronic non-cancer pain. Over the last two decades, opioid prescribing rates and average prescription volumes have increased significantly in a number of regions, including Canada, the United States, Australia, and the United Kingdom [32–34]. There is considerable use of long-term opioid treatment for the management of chronic non-cancer pain, including low back and neck pain. This is particularly true in the United States. For example, between 1999 and 2012, 75% of Medicaid patients with chronic non-cancer pain were prescribed long-acting opioids for chronic back pain [35]. From 1997 to 2009, the likelihood of receiving opioids for back pain in a US emergency department increased at a rate of 35% every five years [36]. In addition, opioids were prescribed for 18.7% of all emergency department discharges from 2006 to 2010, of which neck pain was one of the most common pain conditions for the prescription (51.6%) [37]. Thus, evidence suggests that opioid prescribing for low back and neck pain is prevalent and increasing over time.

### Opioid misuse and chiropractic practice

Back and neck complaints are common presentations in chiropractic clinics [38, 39]. Therefore, chiropractors can play an important role in helping address the opioid crisis by providing safe and effective non-pharmacological interventions for these conditions. Evidence-based clinical practice guidelines recommend the consideration of education, exercise, and spinal manipulation or mobilization as non-invasive interventions for low back and neck pain—interventions commonly provided by chiropractors [40–43]. As a member of the patient’s healthcare team, chiropractors can serve as a valuable resource for musculoskeletal spinal pain management in individuals who cannot or prefer not to undergo opioid drug therapy. This may include co-management of musculoskeletal conditions among individuals at high risk of addiction or relapse of addiction, those currently undergoing addiction rehabilitation programs, pregnant women and individuals at high risk for adverse effects.

The opioid misuse crisis creates an impetus for chiropractors and chiropractic organizations to collaborate with other healthcare providers, decision makers, and stakeholders. Patient centered, inter-professional collaboration should be expanded for the treatment of musculoskeletal pain, with chiropractors playing a larger role on multidisciplinary pain management teams. The WFC commits to supporting initiatives to address the opioid crisis at the local, national and international level. Many initiatives focus on information dissemination to provide healthcare providers with resources to improve non-opioid pain management, raise public awareness about the risks of opioid misuse, and empower people to make safe pain management choice [44–46]. By engaging in these initiatives, the chiropractic profession can demonstrate its value as a part of the broader healthcare community, and its contribution to addressing musculoskeletal pain in a collaborative, evidence-based manner.

## Priority area: Women’s, Children’s and adolescents’ health

### Context

The implementation of United Nations Millennium Development Goals [47] has profoundly impacted the lives of women and children. Both maternal and child mortality have halved since 1990 [47]. It is widely recognized that societies can be transformed when women, children and adolescents not only survive, but also thrive [48, 49]. WHO’s Global Strategy for Women’s, Children’s and Adolescents’ Health 2016–2030 aims to deliver meaningful, measurable outcomes that improve health throughout the world [50]. By targeting the social, economic, environmental and political causes of poor health among women, children, and adolescents, this initiative aims to improve access to healthcare, education and occupational opportunities.

### Women’s, Children’s and adolescents’ health and chiropractic practice

As primary contact healthcare providers, chiropractors can play a key role in women’s, children’s and adolescents’ health [51–53]. Working at international, national, and community levels, chiropractors can address deficits in access to health services for musculoskeletal conditions, including health literacy education, health promotion, preventative strategies, and hands-on care.

In addition to health inequities that disadvantage women’s access to services, [54] other women’s health issues experienced throughout the life course are particularly relevant to chiropractic practice. First, pregnancy-related low back and pelvic pain, as well as post-partum mechanical spinal disorders are common, and can impede recovery, nursing, and care giving [55]. Second, hormonal changes, dietary factors, and physical inactivity levels are risk factors for osteoporosis the ageing female population, which can result in vertebral body insufficiency, hip, spine and pelvis fractures [56, 57]. This can have serious impact on disability, morbidity and mortality [58]. Third, violence against women and girls gives rise to physical and psychological injury, [59] both of which can be revealed during the clinical encounter. In many jurisdictions, chiropractors and other healthcare providers are legally required to report abuse to authorities, and everywhere they have an ethical obligation to support patients experiencing abuse in a culturally relevant manner. Through the provision of treatment, advice, and referrals, chiropractors can employ a range of services to help address these issues.

As major healthcare decision makers within families, women have significant influence on the health of children, from birth to adolescence [60]. This includes dietary habits and nutrition, cleanliness and safety of the home environment, and accompaniment to healthcare services. In many low- and middle-income countries, care also includes that of ageing relatives [61]. Multi-generational aspects of family health care can bring about varying challenges as they relate to facilitating mobility and meeting health challenges encountered throughout the life course. Empowering women as health decision makers is therefore fundamentally important to families on an individual level, and influences the health of communities and societies on a macro level [62]. Patient education relative to healthy eating, family physical activity, home safety, and adherence to recommended healthcare services and health promotion strategies should be a routine component of the clinical encounter with women and families. Chiropractors should also maintain referral networks to community support services to support the diverse needs of women, children, and adolescents.

Spinal pain and other musculoskeletal complaints, including mechanical back and neck pain, are not uncommon among children and adolescents [63–67]. Chiropractic management, including education and appropriate physical activity instructions, and co-management of these conditions following evidence-based best practice standards, clinical practice guidelines and treatment protocols may be appropriate [43, 68, 69]. Nutrition, physical activity, and tobacco use influence musculoskeletal health, growth and development [70–72]. Beyond pain and disability, this can also impact access to education and future employment prospects, particularly in low-income countries. As spinal health experts, [73] chiropractors possess a range of opportunities to participate in public health interventions targeting youth.

## Conclusions

The WFC commits to promoting and facilitating public health strategies for chiropractors to implement in practice. Healthy ageing, opioid misuse, and supporting women’s, children’s and adolescents’ health are priority areas of initial focus. This work builds on the shared goal of primary care and population health, through the prevention of illness, promoting health, improving patient care, and addressing contextual factors in a collaborative and evidence-based manner. Future work in public health for the chiropractic profession should also focus on broader roles such as community engagement and the creation of sustainable systems, engaging key stakeholders locally and globally [74].

As an initial step, the WFC is developing toolkits, with the goal of empowering chiropractors and WFC member organizations to engage in public health activities in the three identified priority areas. Content will include global and regional synopses of the public health concern, discussion points demonstrating the value of chiropractic engagement in these areas, and practical, evidence-based materials that support chiropractors to effectively address these issues with patients in daily practice. In addition, the WFC looks forward to disseminating and supporting the work of member organizations in the identified priority areas, leveraging the knowledge gained from successful initiatives to implement in other areas and communities. Through this type of knowledge exchange and coordination, chiropractic can meaningfully contribute to public health worldwide and be of greater service to our patients and communities.

## Box 1 The WFC Position on Healthy Ageing

The World Federation of Chiropractic (WFC) recognizes the value that older people contribute to society in many important ways and that the extent of this contribution is strongly influenced by the health that older people experience.

Chronic non-communicable diseases, including back pain, neck pain and other musculoskeletal disorders, account for significant burden of disease among older people. Measures that contribute to healthy ageing and address this burden can add tremendous societal value.

With a rapidly ageing global population, the WFC supports the need for a clear and comprehensive public health strategy that responds to the needs, capacities, and aspirations of older people and the changing contexts in which they live.

The WFC strongly endorses the recommendations of the World Health Organization, contained

in its World Report on Ageing and Health (2015), particularly as they relate to the provision of integrated care that focuses on the needs of older people.

The World Federation of Chiropractic supports the role of chiropractors in maintaining good health and functional independence in older adults, promoting preventative services, and managing musculoskeletal conditions as a member of an older person’s healthcare team.

## Box 2 The WFC Position on Opioid Misuse

The World Federation of Chiropractic (WFC) supports a non-drug, non-surgical first line approach to back pain, neck pain and other musculoskeletal disorders.

Opioid prescription for non-cancer pain has become a complex public health crisis. It can have devastating consequences for individuals, families, and communities. These include addiction, overdose, and death.

The WFC advocates an integrated approach to the opioid crisis. Chiropractors provide alternative care options in the management of back pain, neck pain and other musculoskeletal disorders for which opioids are often prescribed.

The WFC supports initiatives involving partnerships between chiropractors and other health care professions, funders, governments, and other stakeholders to address the opioid abuse crisis in a collaborative and evidence-based manner.

## Box 3 The WFC Position on Women’s, Adolescents’ and Children’s Health

The World Federation of Chiropractic (WFC) recognizes that women, children and adolescents face unique individual and societal health challenges, which differ between low, middle and high-income countries around the world.

These challenges include, but are not limited to, health inequity, cultural and religious beliefs, violence against women and children, infant mortality, sanitation, access to services, and health literacy.

The WFC strongly supports the World Health Organization’s Global Strategy on Women’s, Children’s and Adolescents’ Health (2016–2030), which envisions a world in which every woman, child and adolescent can survive, thrive and reach their full potential.

The WFC also endorses the Every Woman, Every Child movement, which is dedicated to achieving better health for women, children and adolescents around the world. It recognizes that chiropractors can contribute to the goal of universal health coverage by providing equitable, accessible, affordable services that address health needs, promote physical activity and healthy diet, and improve social and emotional wellbeing.

The WFC is committed to gender equality, empowering women to adequately address their and their children’s health needs, and reducing societal barriers that pose a threat to the health and wellbeing of women, children and adolescents around the world.

## Abbreviations

WFC: World Federation of Chiropractic
WHO: World Health Organization
